# Predictors of abnormal cytology among HPV-infected women in remote territories of French Guiana

**DOI:** 10.1186/s12905-017-0493-9

**Published:** 2018-01-24

**Authors:** Antoine Adenis, Valentin Dufit, Maylis Douine, Jerome Ponty, Laure Bianco, Fatiha Najioullah, Odile Kilié, Dominique Catherine, Nadia Thomas, Jean Luc Deshayes, Paul Brousse, Gabriel Carles, Claire Grenier, Vincent Lacoste, Vincent Molinie, Raymond Cesaire, Mathieu Nacher

**Affiliations:** 1 Centre d’Investigation Clinique Antilles-Guyane, CIC INSERM 1424, Centre hospitalier de Cayenne, Ave des Flamboyants, 97300 Cayenne, French Guiana; 2 Laboratoire de Virologie, CHU de la Martinique, Fort de France, Martinique; 3 Laboratoire d’anatomopathologie, CHU de la Martinique, Fort de France, Martinique; 4 Service de Gynécologie Obstétrique, Centre hospitalier de Cayenne, Cayenne, French Guiana; 5 AGDOC Association de Dépistage Organisé des Cancers de Guyane, Cayenne, French Guiana; 6 Département des Centres délocalisés de prévention et de soins, Centre Hospitalier de Cayenne, 97300, Cayenne, French Guiana; 7 Service de Gynécologie Obstétrique, Centre Hospitalier de l’Ouest Guyanais, Saint Laurent du Maroni, Cayenne, French Guiana; 8 Laboratoire des Interactions Virus-Hôtes, Institut Pasteur de la Guyane, Cayenne, French Guiana; 9 EA 3593, Ecosystèmes Amazoniens et Pathologie Tropicale, Université de Guyane, Cayenne, French Guiana

## Abstract

**Background:**

Cervical cancer prevention using cervical cytology is insufficiently sensitive, a significant proportion of HPV-infected women having normal cytology.

The objective of the present study was to try to identify factors associated with abnormal cytology in HPV-infected women living in remote areas of French Guiana.

**Methods:**

A study was conducted in women aged 20–65 years having HPV infections confirmed by HPV DNA detection using the GREINER-BIO-ONE kit. In addition to HPV testing, cytology was performed and classified as normal or abnormal. Demographic and life history variables, and infecting genotypes were compared between the normal and abnormal cytology groups.

**Results:**

None of the demographic and life history variables were associated with cytology results. HPV genotype 53 was significantly associated with absence of cytological abnormalities whereas HPV 52, 58, 16 and perhaps 33 and 66 were independently associated with a greater risk of cytological abnormalities. When grouping HPV genotypes in different species, only species 9 (HPV 16, 31, 33, 35, 52, 58, 67) was significantly associated with abnormal cytology AOR = 5.1 (95% CI = 2.3–11.2), *P* < 0.001.

**Conclusions:**

It was not possible to predict which HPV-infected women will have cytological abnormalities or notfrom anamnesis. In this study HPV 53 seemed more benign than other HPV genotypes. On the contrary, species n°9, containing 5 of the genotypes contained in the nonavalent HPV vaccine, was significantly associated with more cytological abnormalities. HPV testing and vaccination with the nonavalent vaccine should be implemented in these remote parts of French Guiana.

## Background

Cervical cancer follows Human Papilloma Virus (HPV) infection. In French Guiana, the standardized incidence rate of cervical cancer is 30.3 per 100,000 women, thus representingthe second most frequent cancer in females [1] and a significant cause of mortality [2]. These figures show that the epidemiology of cervical cancer in French Guiana, a French territory, is closer to what is observed in developing countries than in France [3]. Current screening practice in French Guiana is annual cervicovaginal cytological testing in women aged 25–65 years of age. Although HPV tests have proven superior to cytology for the detection of cervical cancer, primary HPV testing is not standard practice, instead it is performed after an abnormal cytology result.Efforts to conduct organized screening have been implemented on the coastal part of French Guiana, but not in the interior where remoteness makes it difficult [4]. Between 2012 and 2014, a study was implemented in order to determine the prevalence of HPV infection in women aged 20–65 living in these remote parts of French Guiana. This study found anage-standardized HPV prevalence of 35% and showed that 27.2% of women infected with HPV had normal cytology emphasizing the lack of sensitivity of the current recommended screening strategies [5, 6]. Given that primary HPV testing is not performed in French Guiana, our rationale was that women with abnormal cytology may have had particular characteristics or particular HPV genotypes relative to those with normal cytology. Better understanding why some women have normal or abnormal cytology may help in the advocacy for strategic changes in cervical cancer screening.

### Objective

The objective of the present study wasto try to identify factors associated with abnormal cytology in HPV-infected women.

### Study

#### Study design and setting

The design was a cross sectional study which took place between Dec 2012 and Sept 2014 in the remote villages on the Maroni and Oyapock rivers, which can only be accessed by boat or air.

#### Description of the study population and participant characteristics

The study included women aged 20–65 years living in the remote villages on the Maroni and Oyapock rivers. Hysterectomy, pregnancy >3 months, or never having had sex were exclusion criteria.

#### Sampling of study participants

All women presenting at the health center who wished to participate and fulfilled the inclusion criteria and did not have exclusion criteria. The source population was estimated to be 5 000women in our target age group and our aim was to include at least 300 women in order to be able to measure an HPV prevalence of 30% with a 5% margin of error and a 95% confidence interval.

#### Description of intervention

##### Study conduct

Communication in all the villages sensitized the local populations about this public health problem before beginning the inclusion of patients. Local authorities (administrative and traditional) and health center workers were also informed. Radio messages informed the population of the dates when the study would take place in the village. Women wishing to be screened came to the health center at the planned dates. Samples were stored in a cooler until the end of the mission. The samples were then sent to the Virology laboratory of Fort de France Hospital, in Martinique, where an automated method was used for extraction and genotyping. Sample DNA extraction was performed using a minimum of 2 ml in a liquid phase, followed by PCR, and genotyping using the GREINER-BIO-ONE PapilloCheck® kit discriminating between 24 genotypes among which, HR 16, 18, 31, 33, 45, 51, 52, 53, 58, 59, 66, 68… The kit has a high sensitivity and specificity [7, 8]. The kit also allowed identifying multiple infections. The assay is based on the detection of a fragment of the E1 gene of 24 different HPV-types. After the extraction of viral and human DNA from a cervical smear specimen, a PCR fragment of about 350 bp of the E1 gene is amplified by polymerase chain reaction (PCR)in the presence of a small subset of HPV specific primers. A fragment of the human “house keeping gene” ADAT1 (Adenosine deaminase1) is amplified in the same reactionto avoid false negative results. Then, the amplified products are hybridized at room temperature to specific DNA-probes fixed on the DNA-chip. The bound DNA is fluorescently labelled. Finally, unbound DNA is washed away and the PapilloCheckis automatically scanned, analyzed and evaluated respectively using the CheckScanner and CheckReport software.

When HPV was positive and cytology was negative, a gynecological follow up was recommended to verify if HPV positivity disappeared or if cytological lesions appeared.

#### Data collection and analysis

##### Main jugement criteria

Cytological examination of a cervical vaginal smear was performed in the Fort de France University laboratory (Bethesda 2001 criteria) [9]. Liquid-based cytology used the Thin Prep technique (Hologic®) and Papanicoalou staining (Leica autostainer) and visual screening. Only interpretable results were analyzed. Normal/abnormal cytology was defined by the report by the cytologist. Lesions that led to the “abnormal cytology” conclusion were ASCUS (Atypical squamous cells of undetermined significance), ASCH (Atypical squamous cells cannot exclude HSIL), LSIL (Low-grade squamous intraepithelial lesion), HSIL (High-grade squamous intraepithelial lesion) and glandular anomalies.

The detection of HPV DNA was performed using the GREINER-BIO-ONE kit at the Virology laboratory in Fort de France University Hospital. A short questionnaire was used to collect socio economic and demographic data, gynecological and obstetrical history.

##### Data analysis

The group of women with confirmed HPV infection was analyzed. They were then divided in 2 groups, one with normal cytology, and one with cytological abnormalities. These two groups were compared in terms of demographics, and sexual history, and in terms of infecting-HPV genotypes. Crude odds ratios were calculated and multivariate logistic regression was used to look for independent predictors of cytological abnormalities. Power calculations were performed to determine variable for which the study size may have been insufficient. Data was analyzed using Stata 13.0® (College, Station, Texas).

##### Ethical consideration

All patients received an HPV test and cervical cytology free of charge. All included subjects gave written informed consent. Approval was given by: the Comité d’Evaluation Ethique de l’Inserm (CEEI), approval n° 12–064; the Comité Consultatif sur le Traitement de l’Information en matière de Recherche dans le domaine de la Santé (CCTIRS), n° 12.310; and the Commission Nationale de l’Informatique et des Libertés (CNIL), n° 912,459.

##### Availibility of data and materials

Data can be made available after requesting the authorization from the Commission Nationale de l’Informatique et des Libertés (CNIL, 3 Place de Fontenoy - TSA 80715–75,334 PARIS CEDEX 07).

## Results

Among 601 women with a cervical smear of satisfactory quality, 199 had a positive HPV test.

Among the 61 women with abnormal cervical smears, 52 (85%) had a positive HPV test. Among the cytological anomalies there were 30 ASCUS, 7 ASCH, 18 LSIL, 7 HSIL, and 3 glandular anomalies. One woman had ASCH and LSIL, another had HSIL and glandular anomalies, another had LSIL and glandular anomalies, and one had ASCUS and glandular anomalies. Among the 540 women with normal cervical smears, 147 (27%) had a positive HPV test.

Table 1 shows there were no significant differences betweenHPV-infected women with positive cytology and HPV-infected women with negative cytology regarding the demographic and gynecological history variables. Multiple logistic regression models (data not shown) failed to identify any predictor. Multiple HPV infections were associated with a twofold increase in the crude odds ratio of having cytological anomalies on the cervical smear (Table 2). After adjusting for the HPV genotype, this was no longer significant.

**Table 1:** Cytology results and demographic covariates among women infected with HPV

	Cytology negative		Cytology positive		
	no	%	no	%	OR (95% CI)
Total	147	73 .87	52	26 .13	
Age (years)					
20–29	39	66 .10	20	33 .90	4 .2 (0 .6–.27 .5)
30–39	51	72 .86	19	27 .14	Ref
40–49	26	81 .25	6	18 .75	4 .3 (0 .8–.24 .0)
50–64	31	81 .58	7	18 .42	4 .8 (0 .6–.39 .5)
Education					
never	60	84 .51	11	15 .49	Ref
low	45	65 .22	24	34 .78	2 .1 (0 .5–.8 .9)
intermediate and high	39	69 .64	17	30 .36	1 .6 (0 .3–.8 .0)
missing	3	100	0	0	–
Native language					
maroon languages	69	80 .23	17	19 .77	1 .0 (0 .2–.4 .2)
amerindian languages	47	68 .12	22	31 .88	1 .1 (0 .2–.4 .6)
portugueuse	20	71 .43	8	28 .57	Ref
others	11	68 .75	5	31 .25	1 .1 (0 .2–.7 .5)
Age at first sexual intercourse					
< 15	26	76 .47	8	23 .53	Ref
15–17	46	65 .71	24	34 .29	1 .2 (0 .4–.3 .7)
> = 18	20	74 .07	7	25 .93	1 .5 (0 .4–.6 .0)
Missing	55	80 .88	13	19 .12	–
Number of sexual partners in previous year					
0	14	77 .78	4	22 .22	Ref
1	82	68 .91	37	31 .09	8 .2 (0 .8–.84 .5)
> =2	16	84 .21	3	15 .79	2 .4 (0 .1–.41 .8)
Missing	35	81 .40	8	18 .60	–
Parity					
0–1	26	78 .79	7	21 .21	Ref
2–3	37	67 .27	18	32 .73	1 .1 (0 .3–.4 .3)
4–5	35	72 .92	13	27 .08	0 .8 (0 .2–.3 .8)
> 5	49	77 .78	14	22 .22	2 .1 (0 .4–.11 .2)
Contraceptives					
never	96	76 .80	29	23 .20	Ref
oral	28	75 .68	9	24 .32	0 .7 (0 .2–.2 .4)
other	23	62 .16	14	37 .84	3 .3 (1 .1–.10 .4)

**Table 2:** Relation between HPV genotype and cytology positivity among women with a positive HPV test

	Women with normal cytology (*n* = 540)		Women with abnormal cytology (*n* = 61)		Crude OR (CI 95%)	Adjusted OR (CI 95%)	p
HPV HR genotype	No	%	No	%			
52	14	2.6	13	21.3	3.17 (1.4–7.3)	5.5 (1.8–16.8)	0.002
58	7	1.3	9	14.8	4.19 (1.5–11.9)	11.1 (2.9–43)	<0.001
16	10	1.9	9	14.8	2.87 (1.1–7.5)	7.4 (2.1–26.7)	0.002
31	12	2.2	6	9.8	1.47 (0.5–4.1)	2.7 (0.8–10)	0.12
18	9	1.7	5	8.2	1.63 (0.5–5.1)	2.8 (0.5–14)	0.2
68	18	3.3	3	4.9	0.44 (0.1–1.6)	0.9 (0.2–4.2)	0.9
53	19	3.5	0	0.0			
56	10	1.9	3	4.9	0.84 (0.2–3.2)	1.0 (0.2–5.1)	0.9
51	9	1.7	3	4.9	0.94 (0.2–3.6)	1. 8 (0.3–9.9)	0.5
39	7	1.3	3	4.9	1.22 (0.3–4.9)	4.1 (0.8–20)	0.08
others ^a^	24	4.4	14	23.0	1.89 (0.9–4.0)	4.1 (1.3–13.0)	0.01
Multiple infection	32	62.7	19	37.3	2.06 (1–4.3)	1 (0.3–2.9)	0.9
Single Infection	115	77.7	33	22.3	1	–	

^a^ HPV HR 70/45/66/35/73/33/59/82 – cumulated number inferior to total because of co-infections

**Fig. 1:**
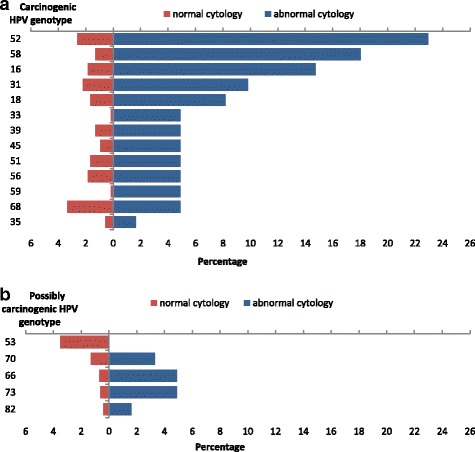
**a** and **b** show the proportion with cytological abnormalities and with normal cytology results by infecting HPV genotype for carcinogenic HPV genotypes and possibly carcinogenic HPV genotypes

Fig. 1a & b and Table 2 show the differences for different HPV genotypes regarding cytological abnormalities. HPV53 was always associated with a normal cervical cytology. Given the absence of HPV53 in women with cytological abnormalities, logistic regression could not be performed. The crude odds ratio was 0 (95% CI0–0.5), *P* = 0.006.

On the contrary, among HPV genotypes identified, HPV 52, 58, 16 and perhaps 33 and 66 were significantly associated with a greater odds of abnormal cytology. Genotypes found in less than 10 women were grouped in another category to run a multiple logistic regression model. Table 2 shows that HPV 52, 58, 16 and “others” (HPV 70, 45, 66, 35, 73, 33, 59, 82) were associated with increased odds of having cytological lesions. For HPV genotypes 31, 18, 68, 53, 56, 51 and 39 the calculated power to detect observed proportion differences between groups was low. Because HPV genotypes are grouped in different species we grouped genotypes in their respective species 5, 6, 7, 9 and 11 [10]. This model showed that only viruses from species 9 (HPV 16, 31, 33, 35, 52, 58, 67) were associated with an increased odds of cytological abnormalities (Table 3). For HPV species 5, 6 and 11 the calculated power to detect observed proportion differences between groups was low.

**Table 3:** Relation between HPV species and cytology positivity among HPV-infected women

	Women with normal cytology (*n* = 540)		Women with abnormal cytology (*n* = 61)		Crude OR (CI 95%)	Adjusted OR (CI 95%)	p
HPV species (genotypes)	No	%	No	%			
5 (51/82)	11	2.0	4	6.6	1.0 (0.3–3.4)	1.7 (0.4–6.7)	0.447
6 (53/56/66)	32	5.9	6	9.8	0.5 (0.2–1.2)	0.6 (0.2–1.6)	0.304
7 (18/39/45/59/68/70)	42	7.8	16	26.2	1.1 (0.6–2.2)	2.2 (0.9–5.1)	0.076
9 (16/31/33/35/52/58)	46	8.5	33	54.1	3.8 (2.0–7.4)	5.1 (2.3–11.2)	0.000
11 (73)	3	0.6	2	3.3	1.9 (0.3–11.8)	1.2 (0.1–9.9)	0.859

HPV 18 was not significantly associated with cytological abnormalities but the number of positive samples may have led to insufficient statistical power.

## Discussion

Among HPV-infected women, 27.2% had a normal cervical smear. This low sensitivity of smears as a screening method is a problem in a remote area where women may not have regular gynecological follow up. The normal demographic or life history variables were unable to predict whether cytology was normal or not. Differences in HPV genotypes, however, were significantly associated with having abnormal cytology. Thus, in our sample, HPV 53 was always associated with normal cytology whereas HPV 52, 58 and 16 were associated with abnormal cervical smears. This may have reflected differences in the number of positive samples for these viruses, with rare genotypes having insufficient numbers for meaningful statistical comparisons. The results may also reflect heterogeneous potential of different HPV genotypes to lead to cytological anomalies [11]. Indeed HPV species 9 (HPV 16, 31, 33, 35, 52, 58, 67) was the only one associated with cytological anomalies. This is of interest since the recently released nonavalent vaccine includes 5 of these viruses (16/31/33/52/58).Multiple HPV infections were associated with cytological abnormalities in the univariate analysis, but not in the multivariate analysis taking into account the HPV genotypes. In fact recent studies performing deep sequencing of HPV E6/E7 genes have revealed loss of genotypic diversity and clonal dominance in high-grade intraepithelial lesions of the cervix. The differences in carcinogenic potential among various HPV genotypes are related tothe evolutionary distances between HPV species (measured using HPV L1 or E6/7 genes) which correlate with the level of IARC-defined carcinogenicity [12, 13].

HPV species 6, which includes HPV 53, is reported to be associated with high risk lesions [14, 15] but also with benign lesions [10, 16]. Although it has been suggested that it had possible oncogenic potential [17], other authors suggested it was a low oncogenic potential [15, 18, 19]. The present results showing an absence of cytological abnormalities suggest a more benign virus than other high risk viruses.

Given the relatively low sample size and the large number of different genotypes, the different cytological anomalies, further studies should compile additional data to give a more robust estimation of the differences between genotypes and HPV species.

Overall, common demographic and life history variables could not predict which women have a higher risk of having positive or negative cervical cytology. On the contrary, different high risk HPV genotypes,notably those from HPV species 9, seemed to have different potentials to cause cytological abnormalities. Vaccination with the nonavalent vaccine thus seems preferable. Presently, given the large proportion of the population that does not have health insurance in these remote areas, the cost of the vaccine is prohibitive. These financial barriers should be removed to achieve sufficient HPV vaccination coverage. Finally, given the remoteness of the villages where the study was conducted and the difficulties of access to care and the low sensitivity of cervical smears alone, future screening strategies should rely on HPV tests alone or in combination with cytology, which have demonstrated their operational interest [20–22].
